# AMPK-Regulated and Akt-Dependent Enhancement of Glucose Uptake Is Essential in Ischemic Preconditioning-Alleviated Reperfusion Injury

**DOI:** 10.1371/journal.pone.0069910

**Published:** 2013-07-26

**Authors:** Lele Ji, Xing Zhang, Wenchong Liu, Qichao Huang, Weidong Yang, Feng Fu, Heng Ma, Hui Su, Haichang Wang, Jing Wang, Haifeng Zhang, Feng Gao

**Affiliations:** 1 Department of Physiology, Fourth Military Medical University, Xi’an, China; 2 Experiment Teaching Center, Fourth Military Medical University, Xi’an, China; 3 Department of Nuclear Medicine, Xijing Hospital, Fourth Military Medical University, Xi’an, China; 4 Department of Cardiology, Xijing Hospital, Fourth Military Medical University, Xi’an, China; Virginia Commonwealth University, United States of America

## Abstract

**Aims:**

Ischemic preconditioning (IPC) is a potent form of endogenous protection. However, IPC-induced cardioprotective effect is significantly blunted in insulin resistance-related diseases and the underlying mechanism is unclear. This study aimed to determine the role of glucose metabolism in IPC-reduced reperfusion injury.

**Methods:**

Normal or streptozotocin (STZ)-treated diabetic rats subjected to 2 cycles of 5 min ischemia/5 min reperfusion prior to myocardial ischemia (30 min)/reperfusion (3 h). Myocardial glucose uptake was determined by ^18^F-fluorodeoxyglucose-positron emission tomography (PET) scan and gamma-counter biodistribution assay.

**Results:**

IPC exerted significant cardioprotection and markedly improved myocardial glucose uptake 1 h after reperfusion (*P*<0.01) as evidenced by PET images and gamma-counter biodistribution assay in ischemia/reperfused rats. Meanwhile, myocardial translocation of glucose transporter 4 (GLUT4) to plasma membrane together with myocardial Akt and AMPK phosphorylation were significantly enhanced in preconditioned hearts. Intramyocardial injection of GLUT4 siRNA markedly decreased GLUT4 expression and blocked the cardioprotection of IPC as evidence by increased myocardial infarct size. Moreover, the PI3K inhibitor wortmannin significantly inhibited activation of Akt and AMPK, reduced GLUT4 translocation, glucose uptake and ultimately, depressed IPC-induced cardioprotection. Furthermore, IPC-afforded antiapoptotic effect was markedly blunted in STZ-treated diabetic rats. Exogenous insulin supplementation significantly improved glucose uptake via co-activation of myocardial AMPK and Akt and alleviated ischemia/reperfusion injury as evidenced by reduced myocardial apoptosis and infarction size in STZ-treated rats (*P*<0.05).

**Conclusions:**

The present study firstly examined the role of myocardial glucose metabolism during reperfusion in IPC using direct genetic modulation *in vivo*. Augmented glucose uptake via co-activation of myocardial AMPK and Akt in reperfused myocardium is essential to IPC-alleviated reperfusion injury. This intrinsic metabolic modulation and cardioprotective capacity are present in STZ-treated hearts and can be triggered by insulin.

## Introduction

A brief episode of myocardial ischemia/reperfusion (MI/R) before sustained ischemia, i.e., ischemic preconditioning (IPC), confers myocardial resistance to lethal ischemia/reperfusion (I/R) injury [1]. Most studies have focused on the role of endogenous triggers, signaling cascades and mitochondria in the cardioprotection afforded by IPC [2], [3], [4]. However, our study as well as several others found that IPC’s cardioprotective effect is abolished in insulin resistance-related diseases such as obesity and diabetes as evidenced in both experimental and clinical studies [5], [6], suggesting that IPC-induced cardioprotection may be associated with myocardial metabolism, lipid profiles, cholesterol levels, etc.

In normal conditions, the heart predominantly uses long-chain fatty acid (LCFA, 60–70%) due to the high energy yield per molecule of substrate metabolized [7], [8]. In the condition of myocardial ischemia, the heart switches to anaerobic glycolysis, a more efficient way to produce ATP [9]. But during myocardial reperfusion, fatty acid (FA) oxidation quickly recovers to be the major source of energy with a concomitant decrease of glucose oxidation, which produces deleterious effects on post-ischemic functional recovery [8]. *In vitro* study has demonstrated that stimulation of glucose metabolism inhibits apoptosis in neurons, cancer cells and leukemic T cells [10], [11]. However, whether glucose uptake is changed and contributes to IPC cardioprotection during reperfusion remained unknown. Therefore, our objective was to determine the role of glucose metabolism in IPC-induced cardioprotection during the early reperfusion period *in vivo* and to explore the possibility to protect the diabetic hearts.

## Materials and Methods

### Streptozotocin-induced Insulin-deficient Rats

The experiments were performed in adherence with the National Institutes of Health Guidelines for the Use of Laboratory Animals and were approved by the Fourth Military Medical University Committee on Animal Care. All surgery was performed under sodium pentobarbital anesthesia, and all efforts were made to minimize suffering. Adult male Sprague-Dawley rats, weighing 180–220 g, were subject to a fast of 12 h before injection. Streptozotocin (STZ, 70 mg/kg) was dissolved in 0.01 M citrate buffer and administrated intraperitoneally. The normal group received citrate buffer only. Blood glucose levels were measured 7 days later by a glucose meter (Life-Scan, Milpitas, CA). Only rats with blood glucose levels ≥16.7 mM (300 mg/dl) were considered to be insulin-deficient and then subjected to surgical procedures followed by the experimental protocol (see below).

### Experimental Protocol

The rats were fasted overnight and anesthetized through intraperitoneal administration of 60 mg/kg pentobarbital sodium. Myocardial ischemia was produced by exteriorizing the heart with a left thoracic incision followed by making a slipknot (6–0 silk) around left anterior descending coronary artery, as described previously [12]. The success in coronary occlusion was confirmed by immediate ST elevation on electrocardiogram.

The effects of IPC on myocardial ischemia/reperfusion (MI/R) injury were examined in normal and STZ-treated rats divided into four experimental groups (n = 6/group), i.e., MI/R, IPC+MI/R, STZ+MI/R and STZ+IPC+MI/R. Additionally, normal rats were randomly assigned to one of the other four groups for study of the effects of IPC on cardiac metabolism and cell signaling (n = 6/group): Sham-operated control rats (Sham), MI/R, IPC+MI/R and IPC+MI/R+wortmannin (W). Experiment was initiated after 15 min of recording to achieve a consistent and stable baseline. The animals were subjected to 30 min of coronary occlusion followed by 3 h of reperfusion (MI/R). IPC was elicited with two consecutive 5 min episodes of coronary occlusion, each followed by a 5 min of reperfusion. Wortmannin (15 µg/kg) was administered intravenously 15 min before IPC. The dose of wortmannin has been demonstrated in our previous study [13], [14]. In separate rats, hearts were excised 1 h after reperfusion and the tissue from the area at risk (AAR) was separated, immediately rinsed in ice-cold saline and then rapidly frozen in liquid nitrogen. The frozen hearts were stored at −80°C and used for Western blot analysis.

### Myocardial Functional Assessment

MI/R-induced cardiac dysfunction was continuously monitored before and during the entire MI/R period. A microcatheter was inserted into the left ventricle through the right carotid artery to measure the left ventricular pressure (LVP) continuously. The artery pressure was measured by right femoral artery intubation. Electrocardiogram, heart rate (HR), the artery blood pressure and LVP were simultaneously recorded on a hemodynamic analyzing system (Chengdu Instrument Co., China). Mean arterial blood pressure (MABP), left ventricular developed pressure (LVDP) and the instantaneous first derivation of LVP (+ dP/dt_max_ and − dP/dt_max_) were derived by computer algorithms and continuously monitored throughout the experiment.

### Determination of Myocardial Infarct Size and Apoptosis

At the end of 3 h reperfusion, myocardial infarct size was determined by a double-staining technique and a digital imaging system (infarct area/area-at-risk×100%). Apoptosis was analyzed by TUNEL (terminal deoxynucleotidyl transferase dUTP nick end labeling) assay using an in situ cell death detection kit (Roche Molecular Biochemicals, Mannheim, Germany) as described previously [14]. The caspase-3 activity of cardiomyocytes was measured by using caspase colorimetric assay kits (Chemicon International, Temecula, CA) as described in our previous study [15].

### Determination of Plasma Creatine Kinase, Blood Glucose, Plasma Insulin and Free Fatty Acid

Plasma creatine kinase (CK) activity was measured spectrophotometrically (Beckman DU 640, USA) in a blinded manner at the end of 3 h reperfusion. Blood glucose was measured by a glucose meter (Life-Scan, Milpitas, CA). Plasma insulin concentrations were measured using a commercial radioimmunoassay kit (Beijing North Institute of Biological Technology, Beijing, China). Plasma free fatty acid (FFA) was measured spectrophotometrically (Beckman DU 640, USA). All measurements were assayed in duplicates.

### *In vivo* Positron Emission Tomography (PET) Studies

Myocardial glucose transport was assessed *in vivo* by measurement of the uptake of ^18^F-fluorodeoxyglucose (FDG) in MI/R rats with a dedicated small-animal PET device. Rats were anesthetized and kept at 37°C. At the very beginning of reperfusion, each rat (∼250 g) was injected with 1 mCi FDG in 100 µL 0.9% saline intravenously. The rats were immobilized on the tray and serial dynamic scans (2 min×30 frames) were performed for 1 h. Static FDG images were reconstructed from the dynamic images.

### Gamma-counter Biodistribution Studies

Animals were euthanized 1 h after PET imaging. The heart was rapidly harvested and the tissue from AAR was rinsed in saline solution, and excess liquid was removed. After weighing the AAR tissue, the radioactivity was measured using a well gamma-counter (Cobra Quantum, Packard Instruments Company, Meriden), as described previously [16]. Cardiac FDG uptake was then expressed as the standard uptake value (SUV) obtained by calculating the ratio of myocardial FDG activity to injected dose normalized to the body and heart weights.

### Intramyocardial Injection of GLUT4 siRNA *in vivo*

GLUT4 specific small interfering RNA (5′-CAGAGCUACAAUGCAACUUTT-3′) or scrambled siRNA were designed and synthesized by GenePharma (Shanghai, China). 20 µg of siRNA were diluted in 40 µl vivo-jetPEI™ and 10% glucose mixture and injected into the apex and anterolateral wall of the heart at 3 different points (the similar points in different rats) with a 30-gauge needle in rats. After 48 hours of siRNA injection, the rats were subjected to IPC followed by MI/R. At the end of reperfusion, hearts were excised for the determinations. GLUT4 knockdown was validated by western blot analysis. As shown in Fig. S1, cardiac GLUT4 expression was significantly decreased following siGLUT4 injection.

### Quantitative Real-time PCR

Total RNA was extracted from flash-frozen tissue with TRIzol reagents (Invitrogen, Carlsbad, CA) and reversely transcribed into cDNA with the reverse transcription reagent kit (DRR047A, TaKaRa) according to manufacturer instructions. Thereafter, 50 ng of cDNA were subjected to quantitative real-time PCR using a PCR detection kit (DRR081A, TaKaRa) on a Bio-Rad CFX96 Real-Time Detection System (Bio-Rad, Hercules, CA). GAPDH was used as the internal control. The following primers were used: GLUT4, forward 5′-CCGGGACACTATACCCTATTCA-3′; reverse 5′-AAGGACCAGTGTCCCAGTCA-3′; GAPDH, forward 5′-GGCACAGTCAAGGCTGAGAATG-3′; reverse 5′-ATGGTGGTGAAGACGCCAGTA-3′.

### Heart Plasma Membrane Preparation

Heart plasma membrane (PM) fraction was performed as described previously [17]. Ventricle tissue was homogenized in buffer A containing (in mmol/L, pH 7.0): 10 NaHCO_3_, 5 NaN_3_, and then centrifuged at 7000×g for 20 min. The pellet was resuspended in buffer B (10 mmol/L Tris-HCl, pH 7.4), and centrifuged at 200×g for 20 min. The supernatant was gently layered on top of a 20% (vol/vol) Percoll gradient in buffer C (in mmol/L: 255 sucrose, 10 Tris-HCl (pH 7.4), 2 EDTA) and centrifuged at 55,000×g for 1 h. The band at density of 1.030 was aspirated and pelleted by centrifugation at 170,000×g for 1 h and resuspended in buffer C as PM solution. Protein concentration of PM solution was determined with BCA protein assay. Glucose transporter 4 (GLUT4) content in PM was determined by Western blot.

### Western Blot Analysis

The protein expression and phosphorylation were measured using Western blot as described previously [18]. The immunoblots were probed with anti-GLUT4 (H-61) (Santa Cruz Biotechnology), anti-phospho-(p)Akt(Thr308), anti-Akt, anti-glycogen synthase kinase3β (GSK3β), anti-pGSK3β (Ser9), anti-AMP-activated protein kinase (AMPK), anti-pAMPK (Thr172), anti-acetyl-CoA carboxylase (ACC) and anti-pACC(Ser79) antibodies (Cell Signaling Technology) overnight at 4°C followed by incubation with the corresponding secondary antibodies at room temperature for 1 h. The blots were visualized with ECL-plus reagent. GAPDH or β-tubulin were used as the internal loading control.

### Statistical Analysis

All values are presented as means ± SEM. Differences were compared by one-way analysis of variation (ANOVA) or repeated-measures of ANOVA followed by Bonferroni correction for post hoc *t*-test. Probabilities of<0.05 were considered to be statistically significant. All of the statistical tests were performed with the GraphPad Prism software version 5.0 (GraphPad Software, San Diego, CA).

## Results

### IPC Failed to Protect STZ-treated Hearts from Ischemia/Reperfusion

After a single STZ injection, >80% of rats were hyperglycemic. The average body weights were 262.83±2.81 and 190.62±5.19 g in control and STZ-treated rats, respectively. As shown in Table 1, injection of STZ markedly decreased plasma insulin, enhanced blood glucose and increased plasma FFA levels. The mortality of STZ-treated rats in the present study was<3%.

**Table 1:** General characteristics of the experimental animals.

Indexes	Con		STZ-treated rats	
	Baseline	MI/R	Baseline	MI/R
Body weight (g)	262.83±2.81	−	190.62±5.19**	−
Blood glucose (mM)	5.24±1.43	6.30±1.87	23.43±2.15**	26.17±3.74
Plasma insulin (mU/L)	27.13±1.92	25.50±3.57	11.14±1.28**	9.42±3.63
Plasma free fatty acid (mM)	0.86±0.07	1.56±0.14**	1.18±0.09*	2.24±0.28#

The table shows general characteristics of the experimental animals at baseline and following MI/R. STZ, streptozotocin; MI/R, 30 min of myocardial ischemia and 1 h of reperfusion. Values presented are means ± SEM; n = 10/group.

* *P*<0.05,

** *P*<0.01 vs. Con-baseline.

# *P*<0.05, vs. STZ-baseline.

**Figure 1:**
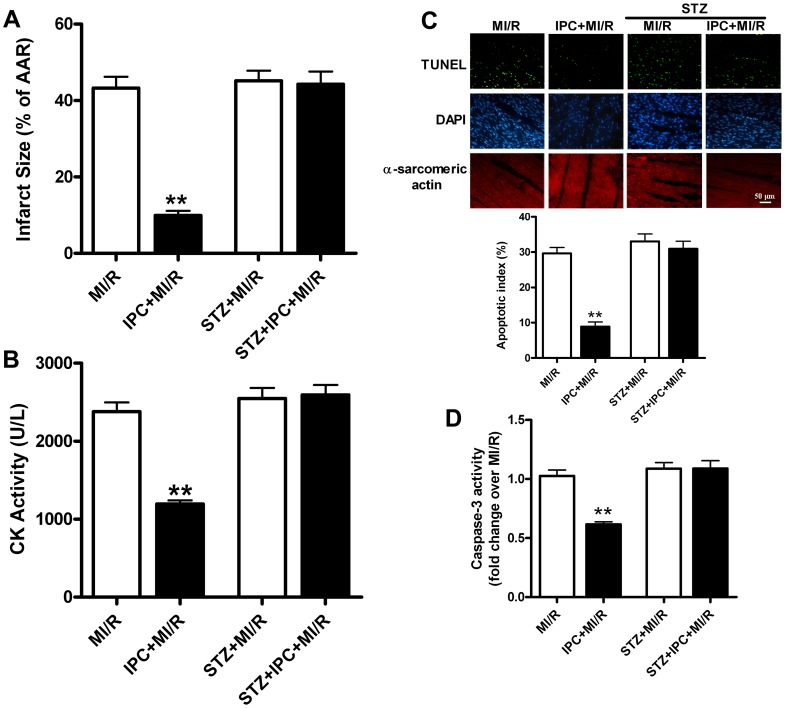
Preconditioning reduces myocardial injury following myocardial ischemia/reperfusion in normal but not STZ-treated rats. (A) Myocardial infarct size (INF) expressed as percentage of area at risk (AAR); (B) Plasma creatine kinase (CK) level; (C) Top: Representative photomicrographs of in situ detection of apoptotic myocytes by TUNEL staining; Green fluorescence shows TUNEL-positive nuclei, blue fluorescence shows nuclei of total cardiomyocytes; Original magnification×400, scale bar represents 50 µm; Bottom: Percentage of TUNEL-positive nuclei in heart tissue sections; (D) Myocardial caspase-3 activity. All rats were subjected to 30 min coronary occlusion followed by 3 h of reperfusion (MI/R). IPC was induced by 2 cycles of 5 min of ischemia/5 min of reperfusion. Streptozotocin (STZ, 70 mg/kg) was administrated intraperitoneally 1 week prior to the surgical procedure. Values presented are means ± SEM; n = 6/group.**P<0.01 vs. MI/R.

**Figure 2:**
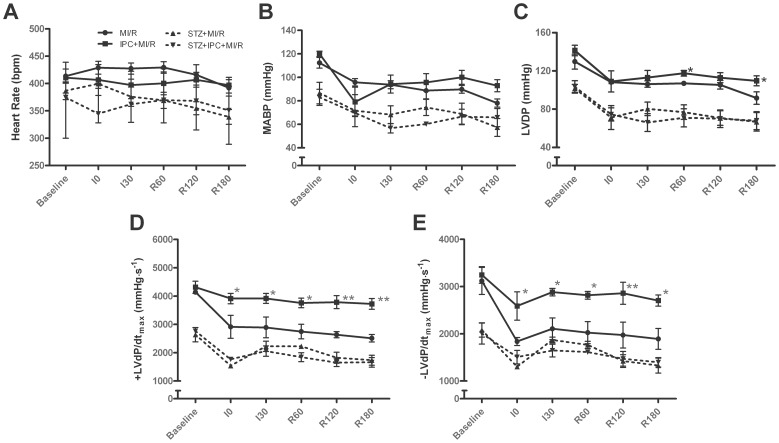
Preconditioning improves cardiac function following myocardial ischemia/reperfusion in normal but not STZ-treated rats. (A) Heart rate (HR); (B) Mean arterial blood pressure (MABP); (C) Left ventricular developed pressure (LVDP); (D, E) Positive and negative maximal values of the instantaneous first derivative of left ventricular pressure (± dP/dt_max_). Values presented are means ± SEM; n = 6/group.*P<0.05,**P<0.01 vs. MI/R. Abbreviations were denoted in Figure 1.

Preconditioning markedly reduced infarct size, plasma CK activity and myocardial apoptosis compared with the MI/R group (all *P*<0.01, Fig. 1) in normal hearts whereas STZ treatment abolished all these beneficial effects of IPC. Consistently, IPC significantly improved myocardial functions during reperfusion as evidenced by increased LVDP,+LV dP/dt_max_ and -LV dP/dt_max_ in normal hearts although there were no significant differences in HR and MABP (*P*<0.05, Fig. 2). However, its improvement of cardiac functions were significantly blunted in STZ-induced insulin-deficient rats.

### IPC Enhanced Glucose Uptake During Post-ischemic Reperfusion in I/R Hearts

**Figure 3:**
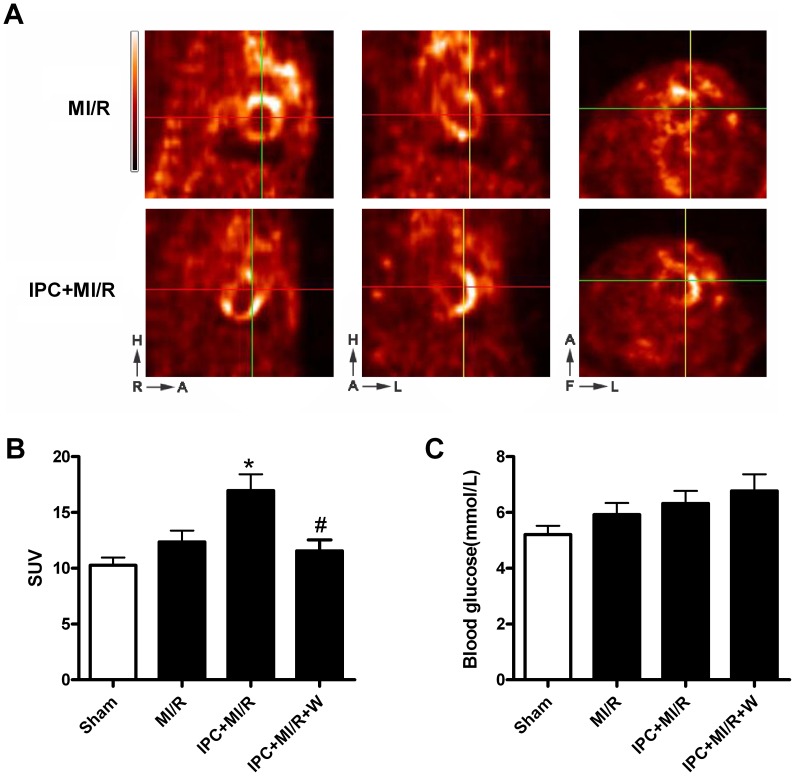
Preconditioning enhances glucose uptake during post-ischemic reperfusion in I/R hearts. (A) Representative positron emission tomography images obtained in rats subjected to MI/R with or without IPC; Top: Sagittal, coronal and transverse images of FDG uptake in MI/R group, respectively; Bottom: Sagittal, coronal and transverse images of FDG uptake in IPC+MI/R group, respectively; At the lower-left corner of the images, A, H, F, R and L indicates anterior, head, foot, right and left, respectively; (B) Bar graphs summarizing the mean data of FDG uptake expressed as standard uptake values (SUVs, y-axis) in vitro gamma-counter tissue biodistribution studies; (C) Blood glucose level; Each rat (∼250g) was injected with 1 mCi FDG in 100 µL 0.9% saline intravenously at the very beginning of reperfusion (n = 12/group). All rats were subjected to 30 min of coronary occlusion followed by 1 h of reperfusion. IPC was induced by 2 cycles of 5 min of ischemia/5 min of reperfusion. Sham-operated control rats (Sham) underwent the same surgical procedures with the exception of left anterior descending coronary artery occlusion. Values presented are means ± SEM.*P<0.05 vs. MI/R, *^#^P*<0.05 vs. IPC+MI/R.

As shown in Fig. 3C, there were no significant differences in blood glucose concentrations among all groups after 1 h of reperfusion, indicating IPC has no effect on systemic glucose metabolism. In addition, it seems plasma insulin levels were slightly increased in IPC group, however, there was no significant difference compared with MI/R group (Fig. S2). To examine whether IPC increases myocardial glucose uptake during reperfusion, we measured myocardial accumulation of the positron-emitting glucose analogue FDG. PET imaging and gamma-counter biodistribution studies were performed. Representative examples of PET images (Fig. 3A) revealed that IPC plus MI/R group showed more obvious FDG uptake in the hearts compared with MI/R group. Consistently, the results of biodistribution studies also indicated IPC stimulated glucose uptake in I/R hearts (*P*<0.05, Fig. 3B). These results illustrated that IPC significantly increased myocardial glucose uptake following MI/R. Importantly, pretreatment of the specific phosphoinositide 3-kinase (PI3K) inhibitor wortmannin significantly blocked the metabolic effects of IPC (*P*<0.05), suggesting that PI3K plays a role in the metabolic modulation of IPC.

### Suppressing GLUT4 with GLUT4 siRNA Markedly Reduced the Cardioprotection of IPC

**Figure 4:**
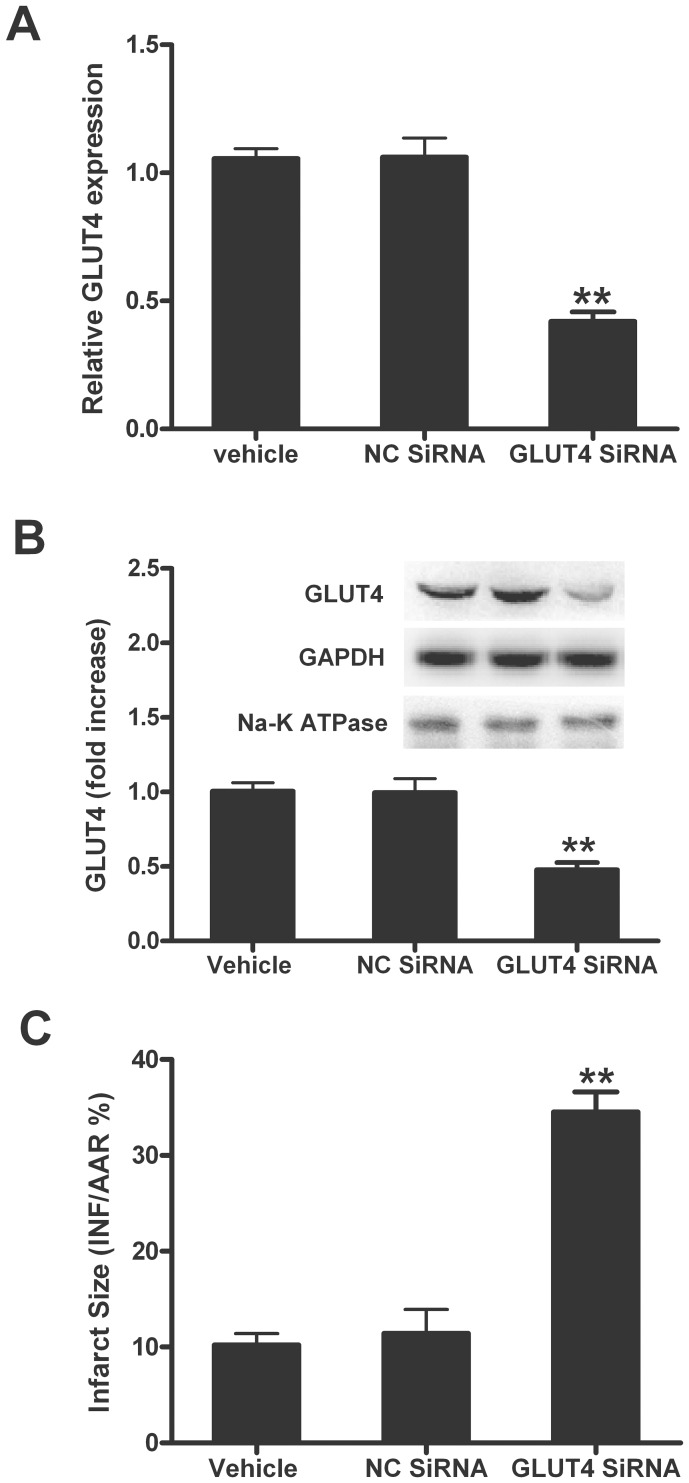
Suppressing GLUT4 with GLUT4 siRNA reduced the cardioprotection of IPC. (A) GLUT4 mRNA expression (n = 4/group); (B) GLUT4 protein expression, Top images: representative blots (n = 4/group); (C) myocardial infarct size expressed as percentage of area at risk (AAR, n = 6/group). All rats were subjected to IPC and 30 min of ischemia followed by 1 h of reperfusion for qPCR and western analysis or 3h of reperfusion for the determination of myocardial injury. IPC was induced by 2 cycles of 5 min of ischemia/5 min of reperfusion. NC, negative control. Values presented are means ± SEM.**P<0.01 vs. NC SiRNA.

We next used a genetic approach to reduce GLUT4 *in vivo* to obtain more solid evidence to support that increased glucose uptake is essential to IPC. After 48 h of intramyocardial siRNA (GLUT4 siRNA or scrambled siRNA) injection, the rats were subjected to IPC and MI/R as described above. As summarized in Fig. 4A and B, cardiac expression of GLUT4 mRNA and protein level was decreased after GLUT4 siRNA injection in rat hearts (*P*<0.01). We next determined whether the reduction of GLUT4 would abolish the protective effects of IPC. As shown in Fig. 4C, myocardial infarct size in GLUT4 siRNA-treated hearts was markedly increased compared with scrambled siRNA-injected hearts (*P*<0.01). Therefore, increased myocardial glucose uptake plays a key role in the cardioprotection of IPC.

### IPC Stimulated Akt and AMPK Activation and thus Increased GLUT4 Translocation in a PI3K-dependent Manner

**Figure 5:**
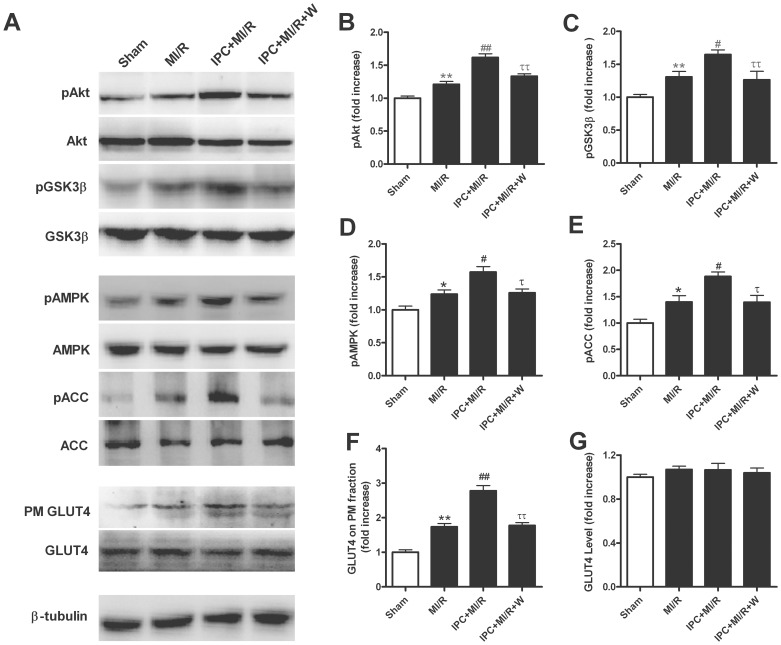
Preconditioning stimulates Akt and AMPK activation and increases GLUT4 translocation in a PI3K-dependent manner. (A) Representative blots; (B) Myocardial phosphorylation of Akt; (C) Myocardial phosphorylation of GSK3β; (D) Myocardial phosphorylation of AMPK; (E) Myocardial phosphorylation of ACC; (F) GLUT4 on PM fraction; (G) GLUT4 expression. All rats were subjected to a 30-min coronary occlusion followed by 1 h of reperfusion. IPC was induced by 2 cycles of 5 min of ischemia/5 min of reperfusion. Sham-operated control rats (Sham) underwent the same surgical procedures with the exception of left anterior descending coronary artery occlusion. Wortmannin (W: 15 µg/kg) was administered intravenously 15 min before IPC. Values presented are means ± SEM. n = 4/group.*P<0.05,**P<0.01 vs. Sham, *^#^P*<0.05, *^##^P*<0.01 vs. MI/R, *^τ^P*<0.05, *^ττ^P*<0.01 vs. IPC+MI/R.

To further investigate the potential mechanisms of action behind IPC-induced myocardial metabolic modulation, we examined the levels of the key cardiac survival factor Akt and the cardiac energy fuel sensor AMPK. As illustrated in Fig. 5, there was no difference in Akt expression among all groups, while pretreatment with IPC significantly increased Akt phosphorylation. Consistent with Akt, IPC markedly increased GSK3β phosphorylation in myocardium in MI/R rats, while there was no difference in total GSK3β expression in all groups. As expected, pretreatment of the specific PI3K inhibitor wortmannin prior to IPC nearly abolished the phosphorylation of Akt and GSK3β. Our results further revealed that IPC treatment markedly increased phosphorylation of AMPK and ACC in I/R hearts (Fig. 5D and E). Interestingly, wortmannin significantly attenuated IPC-induced phosphorylation of AMPK and ACC (*P*<0.05), with little difference in pan protein expression of AMPK among all groups.

We further determined the potential downstream target of Akt and AMPK, GLUT4 translocation, which serves as the rate-limiting step of glucose uptake. Although there was no significant difference in total GLUT4 protein expression among all groups, MI/R significantly increased GLUT4 translocation to PM, and such increase was further enhanced in the preconditioned hearts (*P*<0.05). Importantly, PI3K inhibitor wortmannin significantly inhibited IPC-induced GLUT4 translocation (Fig. 5F). These results suggested that IPC stimulated Akt and AMPK activation and thus increased GLUT4 translocation in a PI3K-dependent manner.

### IPC-induced Cardioprotective Effect was Significantly Reduced when Akt and AMPK-initiated GLUT4 Translocation was Blocked

**Figure 6:**
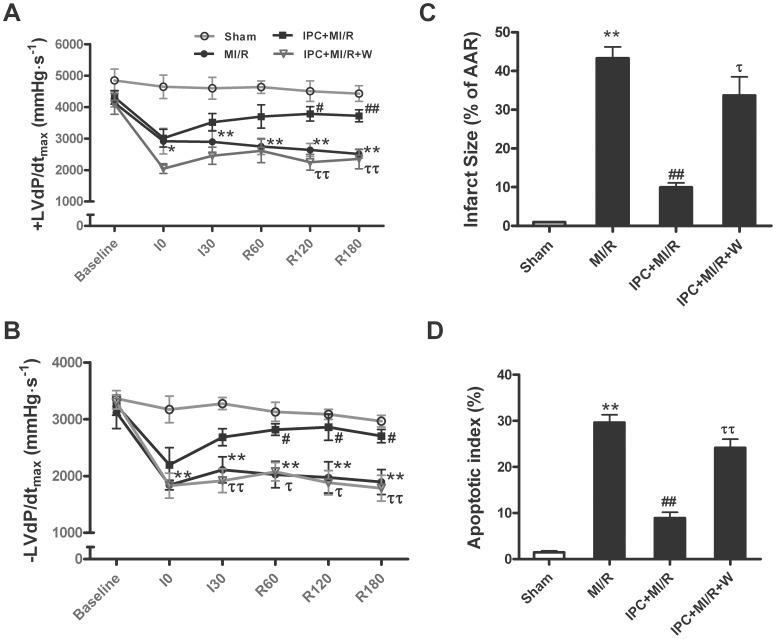
Wortmannin treatment significantly reduced the cardioprotection of ischemic preconditioning. (A, B) Positive and negative maximal values of the instantaneous first derivative of left ventricular pressure (± dP/dt_max_); (C) Percentage of TUNEL-positive nuclei in heart tissue sections; (D) Myocardial caspase-3 activity. Values presented are means ± SEM. n = 6/group.*P<0.05,**P<0.01 vs. Sham, *^#^P*<0.05, *^##^P*<0.01 vs. MI/R, *^τ^P*<0.05, *^ττ^P*<0.01 vs. IPC+MI/R. Abbreviations as in Figure 5.

Having demonstrated that IPC enhanced glucose uptake via a PI3K-dependent and Akt and AMPK-initiated GLUT4 translocation in the ischemic/reperfused heart, we further determined whether blocking IPC-induced metabolic modulation may abolish IPC cardioprotection. There were no significant differences in HR among all groups (Fig. S3A). About 30 min of ischemia followed by 3 h of reperfusion resulted in a significant decline in MABP (Fig. S3B) and ± LV dp/dtmax in I/R group (Fig. 6A and B). IPC significantly increased ± LV dP/dt_max_ compared to the untreated I/R hearts. While application of wortmannin, starting 15 min prior to IPC markedly attenuated the IPC-induced increases of ± LV dP/dt_max_. Meanwhile, administration of wortmannin abolished the IPC-elicited protective effect, with marked increases in myocardial apoptosis index and caspase-3 activity (Fig. 6C and D). Taken together, these data suggest that IPC-induced cardioprotective effect was significantly reduced when wortmannin inhibited Akt/AMPK signaling and subsequent glucose uptake.

### Activation of Akt and AMPK Following Insulin Supplementation Improved Glucose Uptake and Protected the STZ-rats from MI/R Injury

**Figure 7:**
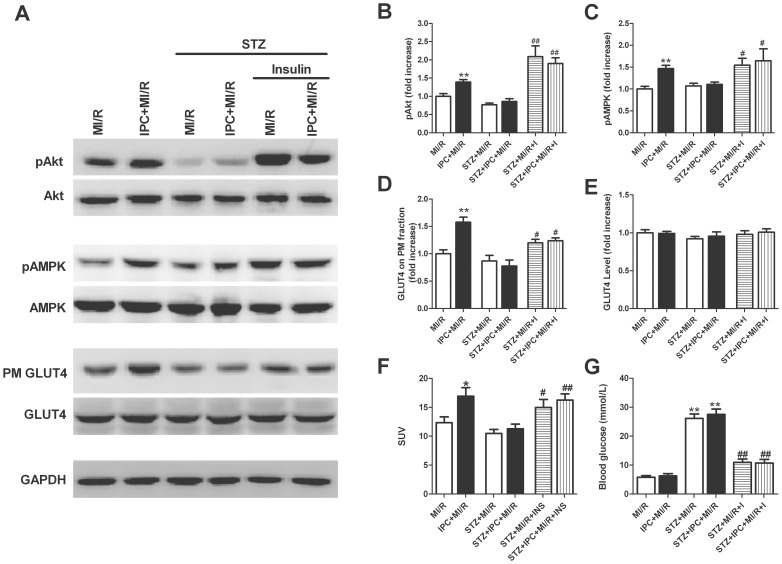
Activation of Akt and AMPK following insulin supplementation improves glucose uptake in the STZ-treated hearts. (A) Representative blots; (B) Phosphorylation of Akt (n = 4/group); (C) Phosphorylation of AMPK (n = 4/group); (D) GLUT4 on PM fraction (n = 4/group); (E) GLUT4 expression (n = 4/group); (F) Data of FDG uptake expressed as standard uptake values (SUVs, n = 6/group); (G) Blood glucose level (n = 6/group). All rats were subjected to 30 min of coronary occlusion followed by 1 h of reperfusion (MI/R). IPC was induced by 2 cycles of 5 min of ischemia/5 min of reperfusion. Streptozotocin (STZ, 70 mg/kg) was administrated intraperitoneally 1 week before surgical procedures. Insulin (I: 60U/L) was infused intravenously at 4 mL/kg per hour starting at 15 min prior to IPC and throughout MI (30 min)/R (1 h or 3 h). Values presented are means ± SEM.*P<0.05,**P<0.01 vs. MI/R, *^#^P*<0.05, *^##^P*<0.01 vs. STZ+MI/R.

To obtain more evidence to support our hypothesis that Akt and AMPK-initiated glucose uptake contributes to the cardioprotection of IPC, insulin supplementation (60 U/L, intravenous infusion at 4 mL/kg per hour, beginning 10 min prior to IPC and throughout MI/R) in the presence or absence of IPC was used in STZ-treated rats. As illustrated in Fig. 7B and C, IPC failed to upregulate Akt or AMPK phosphorylation in the STZ-treated myocardium, while no difference was noted in Akt and AMPK expression. Meanwhile, STZ treatment markedly blunted IPC-induced GLUT4 translocation (Fig. 7D) and glucose uptake (Fig. 7F). However, insulin supplementation significantly increased baseline Akt phosphorylation in STZ rats (Fig. S4). Moreover, insulin treatment alleviated hyperglycemia (Fig. 7G), markedly activated Akt and AMPK and showed a significant increase in GLUT4 translocation and subsequent glucose uptake in ischemia-reperfused STZ hearts with or without IPC. These results favored the notion that augmenting insulin signaling in STZ-treated myocardium plays an important role in Akt and AMPK activation, and these activated kinases significantly alter myocardial metabolism, contributing to the metabolic modulation during reperfusion.

**Figure 8:**
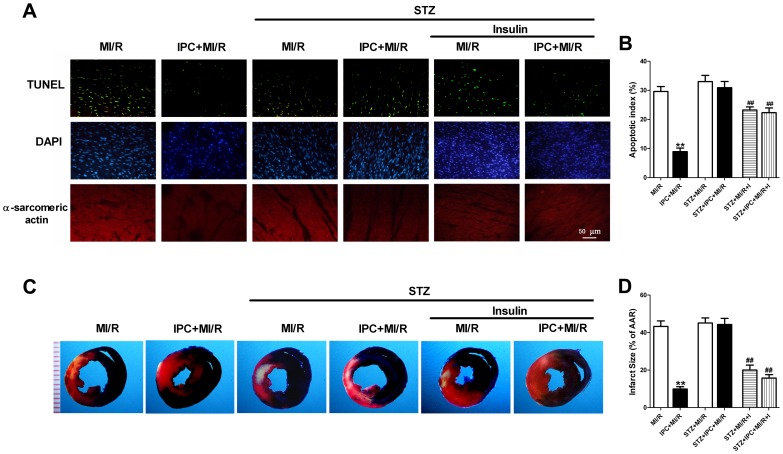
Insulin supplementation protects the STZ-treated hearts from myocardial ischemic/reperfusion injury. (A) Representative photomicrographs of in situ detection of apoptotic myocytes by TUNEL staining; Green fluorescence shows TUNEL-positive nuclei, blue fluorescence shows nuclei of total cardiomyocytes; Original magnification×400, scale bar represents 50 µm; (B) Percentage of TUNEL-positive nuclei in heart tissue sections after 3 h of reperfusion; (C) Representative photographs of heart sections. Blue-stained portion indicates nonischemic, normal region; red-stained portion, ischemic/reperfused but not infarcted region; and negative-stained portion, ischemic/reperfused infarcted region; (D) Myocardial infarct size (INF) expressed as percentage of area at risk (AAR) after 3 h of reperfusion. Values presented are means ± SEM. n = 6/group.**P<0.01 vs. MI/R, *^##^P*<0.01 vs. STZ+MI/R. Abbreviations as in Figure 7.

Consistently, TUNEL assay and myocardial infarct size determination revealed that IPC failed to protect STZ-treated hearts, indicating that inhibition of glucose uptake, at least in part, abolished the cardioprotection of IPC. However, insulin supplementation significantly decreased MI/R-induced apoptotic death and myocardial infarction (Fig. 8). The combination of insulin and IPC did not lead to a further statistically significant increase in cardioprotection. These results further validate that enhanced glucose uptake via co-activation of myocardial Akt and AMPK effectively increased myocardial tolerance to reperfusion injury in the STZ-treated hearts.

## Discussion

The present study investigates the role of myocardial glucose metabolism during reperfusion in IPC with direct genetic modulation, *i.e.*, knockdown of myocardial GLUT4. We found that the reperfused myocardium shows signs of accelerated glucose uptake following IPC and the index ischemia. Wortmannin blocks Akt and AMPK phosphorylation as well as GLUT4 translocation and subsequent glucose uptake, indicating that PI3K-regulated co-activation of Akt and AMPK assumes an important role in IPC-induced metabolic modulation. Importantly, although IPC is abolished in STZ-treated rats due to a failure to increase glucose uptake during reperfusion, the intrinsic metabolic regulation and cardioprotective capacity are present and can be triggered by insulin.

It has been proposed that a shift of myocardial metabolism from FA to glucose oxidation during reperfusion was more oxygen efficient and able to prevent the production of toxic FA intermediates [9]. Previous studies have implicated metabolic abnormalities (FA oxidation provides almost 100% ATP, with a dramatic decrease of glucose utilization) in cardiac contractile dysfunction in STZ-induced diabetes [19]. IPC is a potent form of endogenous protection against myocardial infarction to increase cardiac I/R tolerance. Our data revealed that IPC significantly increased cardiac function and reduced myocardial injury in normal rats, but these beneficial effects were markedly abolished in STZ-induced experimental diabetes. These findings suggest that metabolic disorder contributes to the abrogated cardioprotection of IPC. Since reperfusion leads to accelerated and additional myocardial injury beyond that generated by ischemia alone [20], [21], our study focused on the metabolic regulation of IPC during the reperfusion period following the index ischemia. To test our hypothesis that IPC’s cardioprotection is associated with cardiac metabolism, we first need to investigate whether IPC exerts metabolic modulation. Although experimental evidence has demonstrated that glucose uptake is augmented in canine and rat hearts following the preconditioning trigger (repeated transient non-lethal periods of ischemia and reperfusion) and before the index ischemia [22], [23], little is known about whether IPC afforded metabolic modulation during post-ischemic reperfusion. Previous study has indicated that isolated perfused hearts subjected to IPC showed significantly lower glucose uptake during reperfusion than before ischemia, and preconditioned myocardium has a trend toward decreased glucose uptake during post-ischemic reperfusion [24]. In the present study, we investigated the effect of IPC on glucose uptake *in vivo* using micro-PET and gamma-counter biodistribution. Pentobarbital has been reported to affect the glucose uptake [25]. To exclude the influence of pentobarbital on glucose use, all rats were anesthetized with pentobarbital following the same procedures (60 mg/kg IP injection) for FDG studies, including the experimental and the control groups. To exclude the influence of different circulating glucose levels, all rats were fasted overnight before the experiment. In this situation, IPC has no effect on systemic glucose metabolism whereas preconditioned myocardium showed a significant increase in myocardial glucose uptake during post-ischemic reperfusion. Moreover, IPC significantly improved cardiac GLUT4 translocation in I/R rats. These data strongly support our hypothesis that IPC exerts metabolic modulation during early reperfusion following the index ischemia. More importantly, our *in vivo* study revealed that intramyocardial injection of GLUT4 siRNA significantly decreased GLUT4 expression and thus blocked the cardioprotection of IPC as evidenced by increased myocardial infarct size in IPC-treated I/R hearts, confirming the critical role of glucose uptake in the beneficial effects of IPC.

Insulin is the principal hormone that regulates the uptake of glucose from the blood into muscle and fat cells. In hearts, Akt acts as a key regulator of metabolism and cell survival, and its role in IPC has been well-recognized [2], [26]. Activation of Akt inhibits many pro-apoptotic proteins including BIM, BAX, BAD and p53, ultimately conferring protection against myocardial ischemic injury [27]. In addition, insulin-stimulated glucose uptake in cardiomyocytes is mediated primarily by the PI3K/Akt/GLUT4 pathway [28]. Strong evidence exist that AMPK acts in an additive manner with insulin to increase glucose uptake [29], [30]. This effect of AMPK has been reported to benefit the heart by increasing glucose utilization and subsequently anaerobic ATP synthesis during ischemia [31]. In addition, experimental evidence has demonstrated that activation of myocardial AMPK by metformin following I/R sets into motion events, including endothelial nitric oxide synthase (eNOS) activation, which ultimately lead to cardioprotection [32]. A second major metabolic consequence of AMPK activation during and following ischemia is the stimulation of FA oxidation. Unfortunately, the AMPK-dependent acceleration of FA oxidation occurs at the expense of glucose oxidation, and has the potential to be detrimental in the setting of ischemia/reperfusion [29], [33]. Thus whether AMPK activation is beneficial or harmful to the ischemic heart remains to be completely elucidated. Nishino et al. reported that IPC activated AMPK and up-regulated GLUT4 expression in a PKC-dependent manner [34]. Moreover, AMPK was reported to mediate preconditioning in cardiac cells by regulating the activity and recruitment of sarcolemmal K_ATP_ channels *in vitro* [35]. However, it has yet been unknown whether IPC induced a continued activation of AMPK during reperfusion. Previous study has indicated that Akt interacted with AMPK, orchestrating a bidirectional action on eNOS Ser1179 phosphorylation in H_2_O_2_-treated cells [36]. In addition, experimental evidence has revealed that insulin inactivated AMPK, which was attributable to phosphorylation by Akt of the AMPK α subunits on Ser485 or Ser491 which antagonizes AMPK activation via phosphorylation at Thr172 [37], [38]. Confusing this issue is the observation that AMPK activation is involved in PI3K-mediated palmitate-stimulated glucose uptake in skeletal muscle cells [39]. Therefore, the crosstalk between the two pathways is controversial and the question that whether PI3K-Akt signaling and AMPK signaling cooperates or antagonizes with each other in IPC still remains unclear. Data from the present study revealed that consistent with previous studies [40], [41], Akt phosphorylation was increased in I/R hearts. Since Akt is well-recognized to be cardioprotective, we concluded that in stress, *i.e.* ischemia, Akt activation probably serves as a self-adaptive and compensatory mechanism. IPC significantly enhanced phosphorylation of Akt and GSK3β during reperfusion in MI/R rats, as well as a sharp increase in GLUT4 translocation and subsequent glucose uptake. On the other hand, we observed a significant increase in phosphorylation of AMPK and its downstream target ACC during reperfusion in IPC plus MI/R group, indicating that IPC continuously activated AMPK during reperfusion. These data suggest that under stress conditions, IPC turns the antagonism between Akt and AMPK into the cooperation through increasing ATP production to maintain contractile function and cellular homeostasis. Recent study has indicated that perioperative treatment with glucose-insulin-potassium was associated with a significant reduction in the incidence of low cardiac output state in patients, and this benefit was associated with increased signaling protein phosphorylation including Akt and AMPK [42]. These evidence suggest that the integrated Akt and AMPK activation orchestrated the myocardial functional recovery. Interestingly, our present study showed that as the specific inhibitor of PI3K, wortmannin treatment markedly reduced IPC-induced Akt and AMPK activation, subsequently inhibited GLUT4 translocation, and ultimately abolished the anti-apoptotic effect of IPC. It is well-known that wortmannin inhibits PI3K/Akt signaling. In contrast, neither AMPK nor its upstream kinases are known targets of PI3K. However, recent studies have indicated PI3K inhibitors (wortmannin/LY294002) abolished AMPK phosphorylation in endothelial cells and skeletal muscle cells, suggesting that AMPK activation can be regulated by PI3K [39], [43]. Collectively, these data indicate that in reperfused myocardium, Akt- and AMPK-mediated metabolic modulation co-contributes to IPC-reduced myocardial injury, and PI3K is probably most upstream in the signaling cascade. However, further study is warranted to better elucidate the cross-talk between Akt and AMPK in IPC-elicited cardioprotection against MI/R injury.

To further identify the role of signaling kinases-initiated metabolic modulation in the anti-apoptotic effect of IPC, we determined Akt and AMPK and subsequent metabolic modulation in STZ-treated rats. There were no difference in Akt or AMPK expression among all groups. However, IPC failed to activate cardiac Akt and AMPK in STZ-treated diabetic hearts, accompanying with decreased GLUT4 translocation and reduced glucose uptake, indicating that IPC-induced metabolic modulation disappeared. Previous study demonstrated that insulin used 90 min before the sustained ischemia normalized the blood glucose concentration but failed to restore the cardioprotection of the late phase of IPC in diabetic rabbits (5–6 wk after administration of 100 mg/kg alloxan *i.v.*), and a reduction of eNOS in the aorta could be an explanation for the findings [44]. On the contrary, data from the present study revealed that insulin supplement, starting 10 min prior to IPC and throughout the whole MI/R, effectively recovered the phosphorylation of both Akt and AMPK. In addition, insulin treatment significantly increased myocardial GLUT4 translocation and glucose uptake in STZ-treated hearts. Furthermore, insulin supplement restored the ability of STZ-treated hearts to resist MI/R injury, as evidenced by reduced cardiac infarct size and myocardial apoptosis. We think that activating survival kinases (Akt and AMPK) and thus improving subsequent glucose uptake effectively protect STZ-treated diabetic hearts against MI/R injury. All these provide strong evidence that the intrinsic cardioprotective capacity is present in STZ-rats and can be triggered by insulin. That insulin plus IPC has no additional effects further indicates that Akt and AMPK-initiated metabolic modulation is a major mechanism of cardioprotection.

It is notable that in the present study, STZ was used to induce insulin-deficient rat model. However, STZ-model is a limitation due to its complexity. Rodents injected with STZ not only develop hyperglycemia with decreased insulin level, but show increased serum FFA, cholesterol and triglyceride levels and metabolic dysfunction in hearts. Decreased myocardial function and enhanced oxidative stress are also revealed in STZ animals. Therefore, it is hard to exclude the influence of these confounders. The direct causal relation whereby insulin signaling promotes the metabolic modulation of IPC remains largely associative and hence more speculative. However, data from the present study revealed that insulin signaling, at least in part, contributes to the beneficial effects of IPC. Nevertheless, further studies are clearly needed to clarify the relation between IPC and insulin signaling using more specific animal model.

In conclusion, the present study demonstrates the role of myocardial glucose metabolism during reperfusion in IPC using a novel approach, *i.e.*, direct genetic modulation *in vivo*. Enhanced myocardial glucose uptake during post-ischemic reperfusion contributes to IPC-alleviated reperfusion injury, and that Akt and AMPK activation synergistically mediates the metabolic modulation of glucose in preconditioned myocardium. Furthermore, although IPC is not efficient in STZ-diabetes, the intrinsic cardioprotective capacity is present and can be triggered by insulin.

## Supporting Information

Figure S1 **GLUT4 expression after 48 h of intramyocardial siGLUT4 injection.** Top images: representative blots; Bottom: GLUT4 expression. NC, negative control. Values presented are means ± SEM. n = 4/group.**P<0.01 vs. NC SiRNA.
(TIF)

Figure S2 **Plasma insulin levels.** All rats were subjected to a 30 min coronary occlusion followed by 1 h of reperfusion (MI/R). IPC was induced by 2 cycles of 5 min of ischemia/5 min of reperfusion. Sham-operated control rats (Sham) underwent the same surgical procedures with the exception of left anterior descending coronary artery occlusion. Values presented are means ± SEM; n = 6/group.
(TIF)

Figure S3 **Effects of preconditioning on heart rate and mean arterial blood pressure in rats subjected to 30 min of myocardial ischemia and 3 h of reperfusion.** IPC was induced by 2 cycles of 5 min of ischemia/5 min of reperfusion. Sham-operated control rats (Sham) underwent the same surgical procedures with the exception of left anterior descending coronary artery occlusion. Wortmannin (W: 15 µg/kg) was administered intravenously 15 min before IPC. Values presented are means ± SEM. n = 6/group.**P<0.01 vs. Sham.
(TIF)

Figure S4 **Representative blots of Akt phosphorylation and Akt expression in STZ-treated hearts following insulin supplementation.** All rats were subjected to a 30 min coronary occlusion followed by 1 h of reperfusion (MI/R). IPC was induced by 2 cycles of 5 min of ischemia/5 min of reperfusion. Sham-operated control rats (Sham) underwent the same surgical procedures with the exception of left anterior descending coronary artery occlusion.
(TIF)
