# Supplementary figures and images for: AMPK-Regulated and Akt-Dependent Enhancement of Glucose Uptake Is Essential in Ischemic Preconditioning-Alleviated Reperfusion Injury
Source: PLoS One. 2013 Jul 26;8(7):e69910. doi: 10.1371/journal.pone.0069910 (PMC3724784; doi:10.1371/journal.pone.0069910)

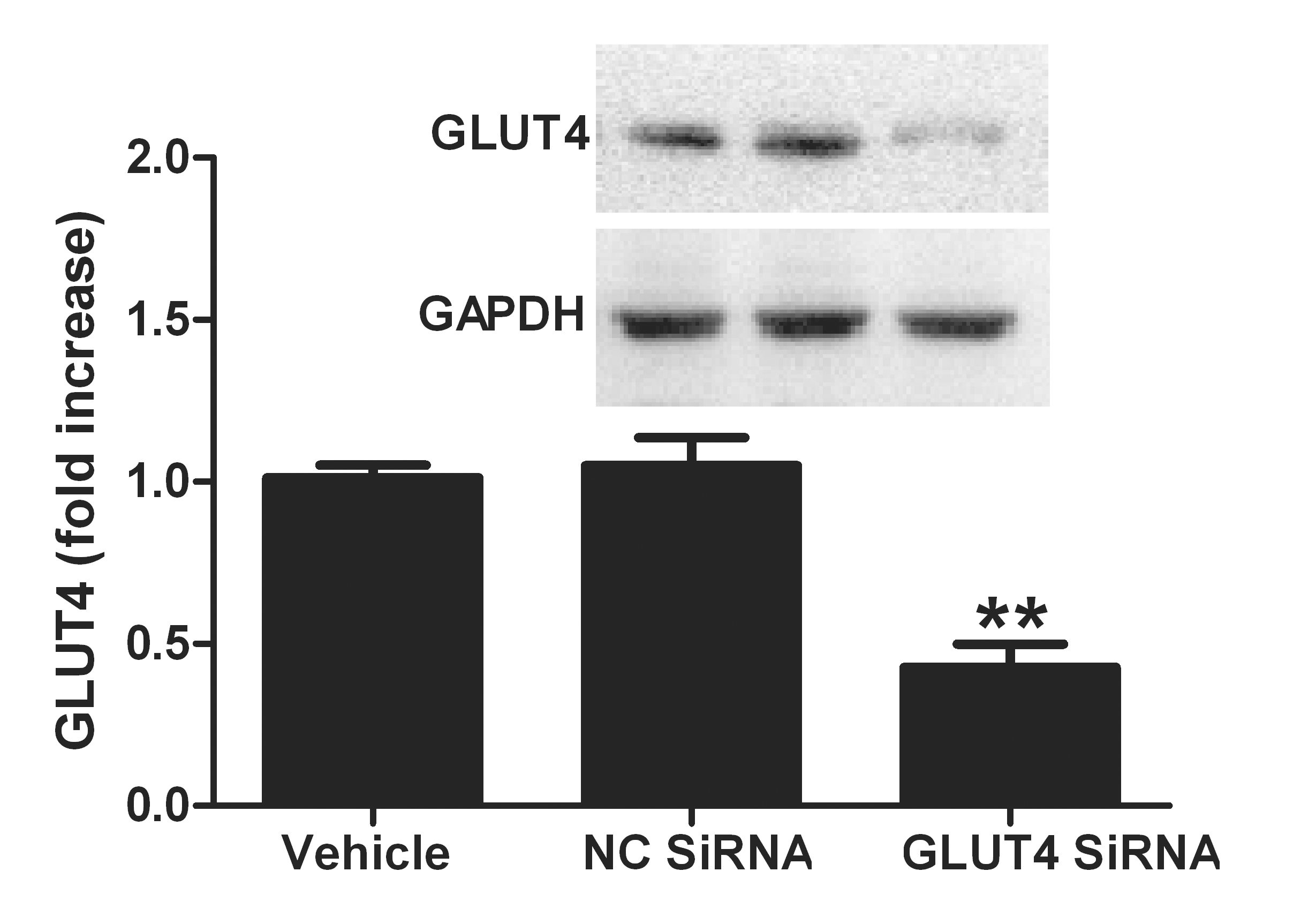

Supplement: Figure S1 — GLUT4 expression after 48 h of intramyocardial siGLUT4 injection. Top images: representative blots; Bottom: GLUT4 expression. NC, negative control. Values presented are means ± SEM. n = 4/group.**P<0.01 vs. NC SiRNA. (TIF) [file pone.0069910.s001.tif]

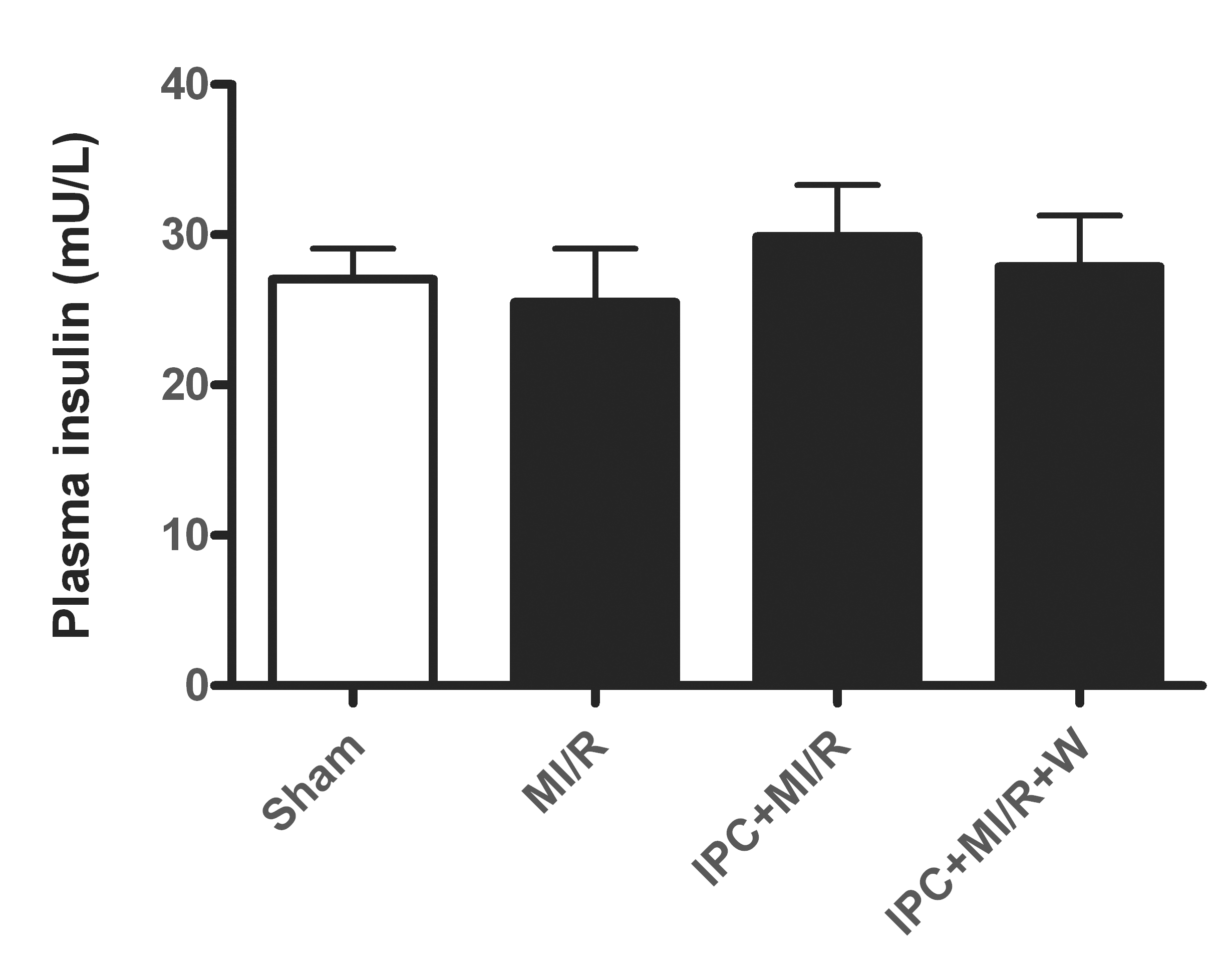

Supplement: Figure S2 — Plasma insulin levels. All rats were subjected to a 30 min coronary occlusion followed by 1 h of reperfusion (MI/R). IPC was induced by 2 cycles of 5 min of ischemia/5 min of reperfusion. Sham-operated control rats (Sham) underwent the same surgical procedures with the exception of left anterior descending coronary artery occlusion. Values presented are means ± SEM; n = 6/group. (TIF) [file pone.0069910.s002.tif]

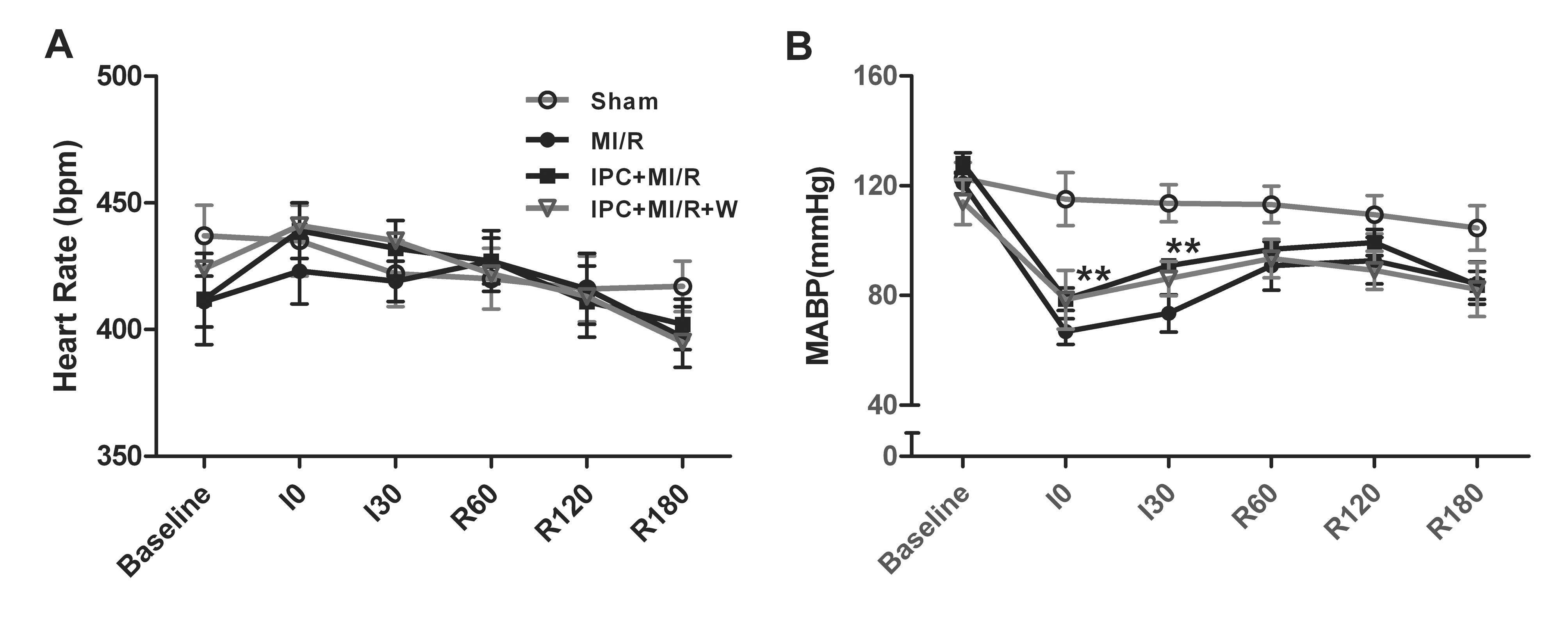

Supplement: Figure S3 — Effects of preconditioning on heart rate and mean arterial blood pressure in rats subjected to 30 min of myocardial ischemia and 3 h of reperfusion. IPC was induced by 2 cycles of 5 min of ischemia/5 min of reperfusion. Sham-operated control rats (Sham) underwent the same surgical procedures with the exception of left anterior descending coronary artery occlusion. Wortmannin (W: 15 µg/kg) was administered intravenously 15 min before IPC. Values presented are means ± SEM. n = 6/group.**P<0.01 vs. Sham. (TIF) [file pone.0069910.s003.tif]

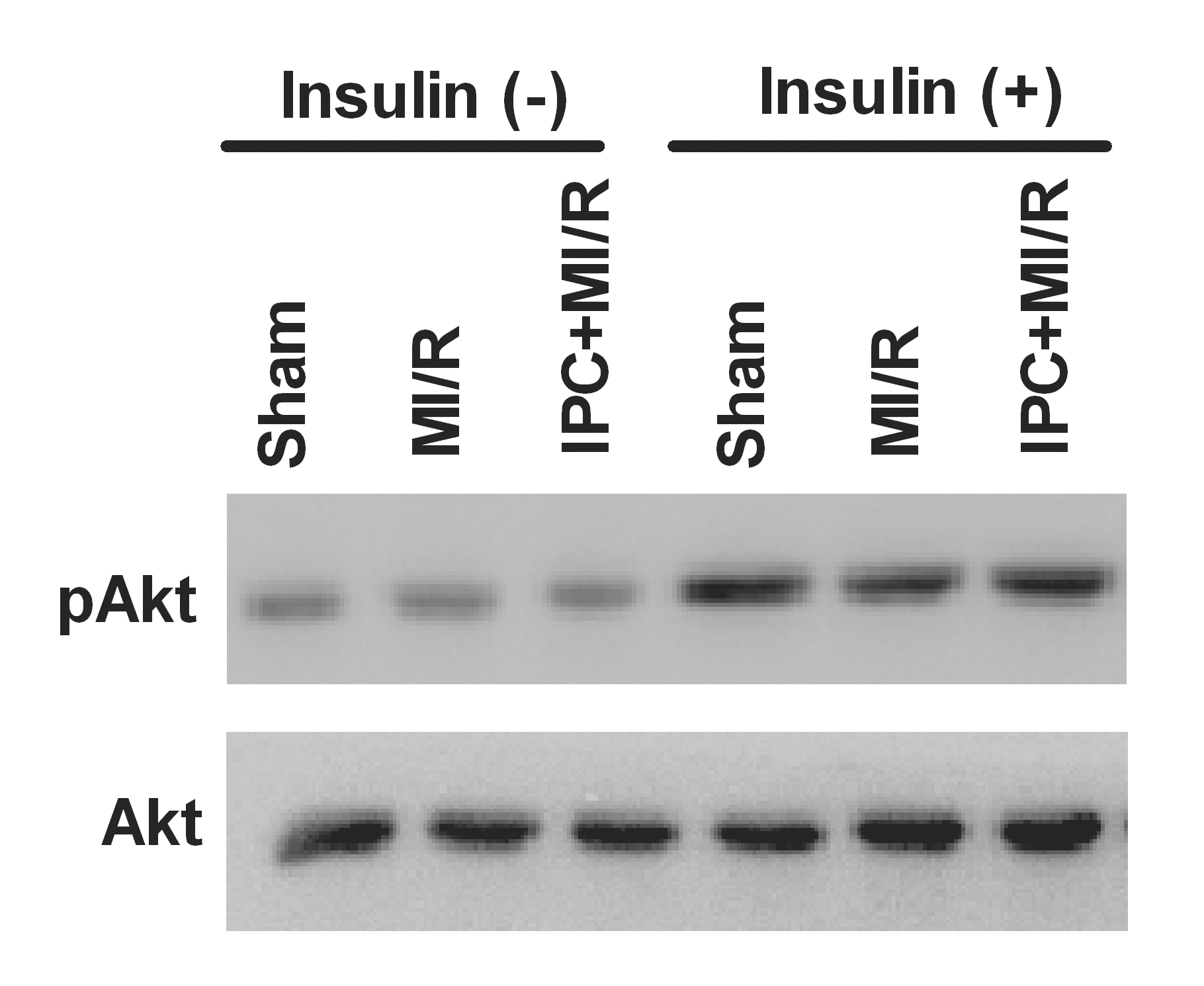

Supplement: Figure S4 — Representative blots of Akt phosphorylation and Akt expression in STZ-treated hearts following insulin supplementation. All rats were subjected to a 30 min coronary occlusion followed by 1 h of reperfusion (MI/R). IPC was induced by 2 cycles of 5 min of ischemia/5 min of reperfusion. Sham-operated control rats (Sham) underwent the same surgical procedures with the exception of left anterior descending coronary artery occlusion. (TIF) [file pone.0069910.s004.tif]
